# Condensation Method for Humidity Measurement in the UMR Cloud Simulation Chamber

**DOI:** 10.6028/jres.093.147

**Published:** 1988-08-01

**Authors:** D. E. Hagen, D. R. White, D. J. Alofs

**Affiliations:** University of Missouri-Rolla Rolla, MO 65401

**Keywords:** aerosol activation, cloud drop growth, expansion cloud chamber, humidity measurement, mixing ratio, water vapor

## Abstract

The University of Missouri-Rolla has developed a cloud simulation facility for the study of various atmospheric cloud processes. The initial relative humidity of the air sample put into the cloud chamber is a key parameter in virtually any experiment and needs to be known accurately. This report describes how the cloud simulation chamber itself has been used as a condensation type hygrometer to calibrate the system’s humidifier. Two distinct and physically different methods for inferring mixing ratio are used, one exploiting the sensitivity of aerosol activation to humidity, and the other exploiting the sensitivity of the rate of growth of cloud droplets to humidity. The two methods give agreement with each other to within a precision of one part per thousand in mixing ratio.

## 1. Introduction

Several countries have developed facilities for accurately measuring humidity. Such a national facility ideally includes both an ultra stable humidity generator, and a primary standard for measuring relative humidity. The primary standard is used to calibrate the humidity generator, and the humidity generator is used to calibrate other portable instruments which have been sent to the national facility for calibration. The gravimetric hygrometer described by Wexler and Hyland [1] utilizes absorption of water vapor by a solid desiccant, and precision weighing of the absorbed water. The uncertainty is about plus or minus one part in a thousand for the measurement of mixing ratio (mass of water vapor per unit mass of dry gas). Gravimetric hygrometers are currently the primary standards in the United States [2], in Japan [3] and in the United Kingdom [4]. A chilled mirror condensation hygrometer [5] is utilized in France as a transfer humidity standard. This instrument is periodically calibrated at the National Bureau of Standards in the United States and then returned to France for use as a standard in France. The accuracy is about plus and minus 0.03 °C in dew point. This corresponds to an uncertainty in mixing ratio of plus or minus 2 parts per thousand, or twice that of the gravimetric hygrometer. The chilled mirror hygrometer however, has the great advantage of portability.

The University of Missouri at Rolla has developed a cloud simulation facility for studying the processes that occur in clouds in the atmosphere. Part of the facility is a humidifier, which has an air flow rate of 1 L/s, and which produces relative humidities close to 100% at temperatures which can be set to within 0.01 °C over the range 5 to 25 °C [6]. Another part of the facility is a cooled wall expansion cloud chamber, which produces a supersaturated environment so that water droplets can be grown on a sample of cloud condensation nuclei (CCN). A third part of the facility is the optical system used to observe the Mie scattering from the water droplets, thus determining the mean size of growing water droplets as a function of time. A fourth part of the facility is the aerosol generation system, used to supply the CCN sample to the simulation chamber. The cloud chamber, optical system, and aerosol generation system, and other components have been described by White et al. [6].

This report describes how the cloud simulation chamber has been used as a condensation type hygrometer to calibrate the humidifier with a precision of one part· per thousand in mixing ratio. An error analysis to determine whether the absolute accuracy is as good as the precision has not been made. Since an error analysis for the gravimetric hygrometer has been made [1], there is more confidence in the absolute accuracy of the gravimetric hygrometer than in our facility. It would therefore be desirable to intercompare a gravimetric hygrometer to our facility, but this direct intercomparison wouldn’t be feasible since neither facility is portable. It would be feasible to compare our humidity measurement technique to a portable hygrometer such as the chilled mirror instrument described by Merigourx and Cretinon [5] and our Center would welcome such an opportunity.

## 2. Expected Humidity from Humidifier

The humidifier consists of two columns each with 60 vertical glass rods, 0.7 cm diameter, spaced on alternate points of a 1.27-cm grid, and enclosed in an aluminum cylinder 16.5 cm inside diameter. Water flows downward on the glass surfaces, and air flows downward in the space between the glass rods. The two humidifier columns, each with an effective length of 89 cm, are connected in series with respect to the air flow. Thus the length of the humidifier is in effect 178 cm. The air flow rate is 1.0 L/s, and the water flow rate is 0.25 L/s.

The first concern addressed was whether the glass rods were isothermal over their length. As water evaporates at the rod surface the rods are cooled due to the latent heat absorbed. To calculate the amount of cooling an energy balance is performed. To humidify originally dry air the latent heat flux is 35.6 watts. This heat when supplied by the 0.25 L/s liquid water flow produces only a 0.034 °C temperature drop in the water. Thus the glass rods have essentially no axial temperature gradients.

The next concern is how to model the vapor diffusion in the humidifier. The problem is three dimensional, and would need to be solved numerically if one wished an accurate analysis. Instead a crude analysis was performed in which the three dimensional problem is approximated as a two dimensional problem for which an analytical solution exists. Specifically, the humidifier is modeled as a round tube, having the same hydraulic diameter as the cross section of the humidifier. The air velocity in the model tube equals the average air velocity in the actual humidifier (5.25 cm/s), and the length of the model tube equals the total length of the two humidifier columns (178 cm). The wall of the model tube is wet and isothermal. The air enters the model tube dry and at the wall temperature. The outlet relative humidity from the model tube is sought.

The solution to the above idealization of the vapor diffusion in the humidifier has been given by Shah [7]. The hydraulic diameter is *4A* / *P*, where *A* is the cross sectional area of the flow and *P* is the perimeter wetted by the flow. Applied to the humidifier, *A* is the area of a 16.5 cm circle minus the area of 60 circles of 0.7 cm diameter. *P* is the circumference of 60 circles 0.7 cm diameter. This gives a hydraulic diameter of 5.66 cm. The Reynolds number based on a 5.66 cm length and an air velocity of 5.25 cm/s is about 200, so the air flow is laminar. The Peclet number is about 115, so that vapor diffusion in the flow direction is small compared to that in the transverse direction. The 178 cm length divided by the 5.66 cm length and by the Peclet number is a nondimensional quantity called x* by Shah. The mean outlet relative humidity, called (1−*θ*_m_) in Shah’s analysis, is a function of only x*. For our x* (0.272) the predicted mean outlet relative humidity is 98.45%, equivalent to a mean outlet dew point lower than the wall temperature by 0.256 °C. This result should be treated as a crude estimate. The resulting predicted dew point deficit of 0.256 °C is an order of magnitude estimate. It turns out that the observed humidity deficit is 0.29 °C, so the agreement is better than one could reasonably expect. Shah also gives an analytical solution for the equivalent diffusion problem between parallel plates. If the humidifier is evaluated in terms of equivalent parallel plates, the predicted dew point deficit is about an order of magnitude smaller (i.e., about 0.03 °C).

## 3. Humidity Measurement in the UMR Cloud Simulation Chamber

The initial relative humidity in the chamber, prior to expansion, is a key parameter in virtually any experiment. Despite the care that has gone into sample humidification, this has proved to be a difficult parameter to fix experimentally, and we have had to enlist additional means over and above the humidifier analysis given above in order to infer the humidity’s value.

Two distinct and novel methods of inferring the initial mixing ratio from Mie scattering on a mono dispersed cloud are described below. These have proved sufficiently accurate and reliable that we have adopted them in all subsequent experiments. The variable chosen to represent the initial water vapor content is mixing ratio, i.e., the number of grams of water vapor per gram of dry air and is denoted by *r*_0_.

### 3.1 Experiment Description

The experiment begins with a moist aerosol laden air sample at temperature *T*_0_ and pressure *p*_0_. Usually *T*_0_ is near 20 °C and *p*_0_ is near 14.1 psi. The aerosol consists of NaCI particles of size near 0.025 *μ*m radius and concentration near 100/cm^3^. The sample’s initial relative humidity is typically near 83%. It is lowered below 100% by immediately raising the sample’s temperature after it leaves the humidifier to avoid condensation loss of vapor (see [6]). At a relative humidity of 83%, the aerosol particles consist of very small (~0.05 *μ*m) solution droplets instead of dry particles. The sample is expanded to give a cooling rate of 10 °C/min, and the resulting cloud is observed via Mie scattering of laser light to determine drop size as a function of time. The aerosol exerts a negligible influence on the measurements of interest here. At our smallest observable drop size, 0.70 *μ*m, a 10% change in the NaCI particle mass would only change the solution drop’s equilibrium supersaturation ratio by 0.000006; an amount negligible in comparison to unity. Small variations in the critical supersaturation of the aerosol would have no appreciable effect on droplet growth rates.

### 3.2 Multiple Droplet Growth Rate Method

The condensational growth of water droplets is quite sensitive to humidity. Hence droplet growth rates can be used to give a measure of the air’s water vapor content. In our method we take a small set of droplet growth rate data containing at least two growth rate measurements and covering a short period of time. This data set is analyzed using drop growth theory to extract two unknowns, mixing ratio and sticking coefficient (*β*). A whole experiment can be subdivided into numerous small data sets, and a separate determination of *r*_0_ made for each one. In each case the amount of water converted into the liquid state is accounted for in the determination of initial (before expansion) mixing ratio *r*_0_. *r*_0_ should of course be constant.

The droplet growth rate equation [8] for the i-th droplet growth rate measurement can be written

$$\begin{array}{l} r_{0}=\epsilon /{\left[p_{i}e_{s}{\left(T_{i}\right)}^{-1}{\left\{(a_{i}+1_{i}){\dot{a}}_{i}/D_{\text{eff}}\rho_{\text{sat},i}+S_{i}*\right\}}^{-1}-1\right]}^{-1}\\ \;+(4/3)\pi Na_{i}^{3}, \end{array}$$ (1)

with ϵ=0.62197, *p* is the total pressure, *N* is the droplet concentration per gram of dry air, *e*_s_ is water saturation vapor pressure, *T* is absolute temperature, *a* is the droplet radius, *ȧ* is the droplet growth rate, *D*_eff_ is an effective diffusion coefficient (see [9]) for water vapor in air, *ρ*_sat_ is the saturation water vapor density over a flat surface, S* is the equilibrium supersaturation ratio over a droplet of radius *a*, and

$$1=D_{\text{eff}}(1_{\alpha }LB/KR_{v}T+1_{\beta }/D),$$ (2)

$$1_{\alpha }=(1-\alpha /2)K(\gamma -1){\left(8\pi T/R_{a}\right)}^{1/2}{\left[\alpha p(\gamma +1)\right]}^{-1},$$ (3)

$$1_{\beta }=(1/\beta -1/2)D{\left(2\pi /R_{v}T\right)}^{1/2}.$$ (4)

Here *D* is the diffusion coefficient for water vapor in air, *L* is the latent heat of condensation for water, *B* is the slope of the *e*_s_ vs temperature curve, *K* is the thermal conductivity of moist air, *R*_v_ is the gas constant for water vapor, *R*_a_ is the gas constant for dry air, *α* is the thermal accommodation coefficient (here we use *α* = 1), and *γ* is the specific heat ratio of moist air. The amount of water in the system is constant, so eq (1) should have the same value for all drop growth rate measurement points. This fact can be used to eliminate the unknown *β*. Define a minimization parameter as:

$$\chi^{2}=\frac{\sum_{i=1}^{I}\sum_{j=1}^{i-1}{\left(r_{0i}-r_{0j}\right)}^{2}/{\left[\delta (r_{0i}-r_{0j})\right]}^{2}}{\sum_{\text{ij}}{\left[\delta (r_{0i}-r_{0j})\right]}^{-2}},$$ (5)

where δ(*r*_0i_−*r*_0j_) denotes the uncertainty in the knowledge of *r*_0i_−*r*_0j_. This uncertainty is calculated based on a 0.05 *μ*m uncertainty in a, a 20% uncertainty in *ȧ*, and a 5% uncertainty in *N*. The condensation coefficient enters into χ^2^ through 1. Since *r*_0_ is constant χ^2^ should be zero or at least small. Using the computer routine STEPIT, *β* can be varied to minimize χ^2^. This yields a value for *β* which can be put back into eq (1) to get *r*_0_. This process can be repeated for each data subset taken during the experiment to give multiple measurements of the same quantity *r*_0_.

### 3.3 Cloud Arrival Time Method

During a given expansion, the cloud is observable soon after saturation (100% relative humidity) is reached, and this observation provides a good determination of the saturation event. The cloud droplets can be observed starting at 0.70 *μ*m radius. The small time increment, on the order of one second, between saturation and first cloud observation, can be accounted for by droplet growth modeling. For the special case of expansions using constant cooling rates (temperature is a linear function of time), this time increment is very insensitive to *r*_0_. The method simply involves observing the cloud arrival time, subtracting the above time increment from this to determine the time at which the gas sample reached 100% relative humidity, noting the gas temperature and pressure at this time (100% relative humidity), and finally calculating the mixing ratio *r*_0_ of the gas from this information.

This method is only applicable to experiments using constant cooling rates which make the above time increment independent of *r*_0_. Also the method is applied only to experiments using monodispersed CCN aerosols and fairly fast expansion rates. These conditions lead to monodisperse clouds (all drops are the same size), and allow the use of Mie scattering of laser light as the drop sizing method [6].

The time increment between the 100% relative humidity point and the cloud first observation point is calculated from droplet growth theory, and does depend on the sticking coefficient. However this particular situation involves freshly produced droplets, and fresh water surfaces are thought to have large sticking coefficients near unity [10]. The sticking coefficient results taken from the droplet growth rate method described above confirm this for the early stage of the cloud’s lifetime. Hence, in the time increment calculation, a sticking coefficient of unity is assumed. The influence of sticking coefficient is modest here in any case. A change in sticking coefficient by a factor of 2 (or 5) leads to a change in *r*_0_ by an amount 0.000006 (or 0.000023).

The cloud arrival time method hasn’t been optimized and therefore improvements could be achieved. For instance, the influence of the sticking coefficient could be minimized by choosing a slower expansion rate and a more sharply monodispersed CCN aerosol. This would reduce the change in temperature, pressure, and humidity during the time interval during which the cloud drops grew to observable size.

### 3.4 Numerical Cloud Model Tests

As discussed above, a numerical cloud model, which calculates gas thermodynamics and water droplet growth processes, is used in this analysis. For this we use the numerical model presented by Hagen [9]. This model has been successfully tested against older results in the literature [9] and against the NASA Analytic Simulator [11,12]. Anderson, Hallett, and Beesley [13] presented an extended solution to the droplet growth problem and gave an intercomparison among the leading droplet theories. They conclude that Carsten’s [8] solution of the droplet growth problem is quite adequate. This is the solution on which our cloud model is based. Furthermore Anderson et al. [13] presented numerical results from their extended model for numerous test cases. We calculated these cases with our model and agreed with their results to within 2 % for drop size.

## 4. Results

The experimental results from five different days of work are given in table 1. The run number gives a unique identification number for each experiment. The first six digits of the run number give the date (month-day-year) on which the experiment was performed. These experiments run from September 1986 to November 1987. The column labeled *r*_0_(MDG) gives the values for the mixing ratio obtained from the multiple drop growth rate method. This represents the average of repeated measurements of this quantity during the course of the experiment. Since *r*_0_ represents the initial value of the mixing ratio, it is fixed and all of these repeated measurements should yield the same value. *σ*(MGR) denotes the standard deviation of the individual results around the average and is a measure of experiment consistency. Note that *σ*(MGR) is usually small, indicating good consistency between the various measurements taken in a given run. *r*_0_(CAT) denotes the initial mixing ratio as determined by the cloud arrival time method. Δ*r*_0_ denotes the difference between the two methods, i.e., Δ*r*_0_*=r*_0_(CAT)−*r*_0_(MGR).

The average of the absolute difference between *r*_0_(MDG) and *r*_0_(CAT) is 0.000010, with standard deviation around the average of 0.000007. This average difference between the two methods corresponds to a difference of 0.012 °C in dew point. Hence the two methods are in good agreement with each other. The average difference between measured humidifier temperature and dew point is 0.29 °C, corresponding to a relative humidity of 98.1 % for the air coming from the humidifier. This figure is in agreement with the rough estimate of expected humidifier performance under these conditions (0.26 °C difference between humidifier temperature and dew point, or 98.45% relative humidity for air leaving the humidifier) that was given above.

## 5. Conclusion

The problem of determining the water vapor content of gas sample is addressed using the condensational growth of airborne water droplets as the measurement tool. Two distinct methods are employed. One is based on multiple measurements of droplet growth rate; the other is based on the fact that cloud is observable almost immediately after the gas sample is brought through 100% relative humidity. These two quite different methods give good agreement with each other. The average difference in calculated initial mixing ratio is 0.000010 grams of water per gram of dry air (corresponding to about 8 parts in 10^4^), with a standard deviation of 0.000007 gram/gram-air. This corresponds to a dew point difference of 0.012 °C. This agreement gives us substantial confidence in our determination of water vapor content.

## Figures and Tables

**Table 1:** Numerical cloud model tests

Run	*r*_0_(MGR)	*σ*(MGR)	*r*_0_(CAT)	Δ*r*_0_
091086.04	0.012582	0.000008	0.012591	0.000009
091086.05	0.012574	0.000012	0.012550	−0.000024
091086.06	0.012580	0.000022	0.012570	−0.000010
091086.07	0.012605	0.000005	0.012615	0.000010
111886.A1	0.012618	0.000010	0.012630	0.000012
111886.A3	0.012733	0.000007	0.012752	0.000019
111886.A4	0.012551	0.000010	0.012565	0.000014
121786.01	0.012480	0.000005	0.012488	0.000008
121786.02	0.012483	0.000007	0.012485	0.000002
121786.03	0.012503	0.000006	0.012512	0.000009
121786.04	0.012521	0.000013	0.012533	0.000012
121786.05	0.012509	0.000006	0.012519	0.000010
121786.06	0.012520	0.000008	0.012524	0.000004
121786.07	0.012530	0.000011	0.012526	−0.000004
121786.08	0.012523	0.000008	0.012541	0.000018
121786.09	0.012525	0.000012	0.012523	−0.000002
121786.10	0.012528	0.000045	0.012524	−0.000004
080787.01	0.012668	0.000018	0.012689	0.000021
100687.01	0.012510	0.000008	0.012506	−0.000004

## References

[1] Wexler A, Hyland RW, Wexler (1965). The NBS standard hygrometer In Humidity and Moisture.

[2] Hasegawa S National basis of accuracy in humidity measurements. Moisture and Humidity, Instrument Society of America.

[3] Takahashi C, Inamatsu T Construction of a gravimetric hygrometer. Moisture and Humidity, Instrument Society of America.

[4] Forton AG, Pragnell RF Development of the primary gravimetric hygrometer for the U.K. national humidity standard facility. Moisture and Humidity, Instrument Society of America.

[5] Merigoux J, Cretinon B A transfer humidity standard for dew point temperatures in the range from −20 °C and +60 °C. Moisture and Humidity, Instrument Society of America.

[6] White DR, Kassner JL, Carstens JC, Hagen DE, Schmitt JL, Alofs DJ, Hopkins AR, Trueblood MB, Alcorn MW, Walker WL (1987). Rev Sci Instrum.

[7] Shah RK (1975). Thermal entry length solutions for the circular tube and parallel plates.

[8] Carstens JC (1979). Adv Colloid Interface Sci.

[9] Hagen DE (1979). J Appl Meteor.

[10] Pruppacher HR, Klett JD (1978). Microphysics of clouds and precipitation.

[11] Plooster M (1979). Atmospheric cloud physics laboratory simulation system; mathematical description.

[12] Plooster M (1979). Atmospheric cloud physics laboratory simulation system; users guide.

[13] Anderson BJ, Hallett J, Beesley M (1981). An extended classical solution of the droplet growth problem.

